# The Quality of Home Support Services for Older People in Ireland and Its Implications for China

**DOI:** 10.1093/geroni/igaf122.2840

**Published:** 2025-12-31

**Authors:** Guolei Zhang, Caifang Zhang

**Affiliations:** University College Cork, Cork, Cork, Ireland; Hangzhou Normal University, Hangzhou, Zhejiang, China (People’s Republic)

## Abstract

**Background:**

As global aging accelerates, home support services (HSS) are essential for enabling older adults to age in place with dignity. Ireland’s well-established HSS system serves as a benchmark for high-quality elderly care. In contrast, China faces challenges such as rural-urban disparities, reliance on informal care, and insufficient caregiving standardized . This is the first study exploring how Ireland’s successful HSS model can improve China’s elderly care service quality.

**Aim:**

This study aims to identify practical strategies from reland’s HSS model that can be adapted to improve the quality and effectiveness of HHS for older people in China.

**Method:**

The SERVQUAL model was applied to evaluate service quality across five dimensions: reliability, responsiveness, assurance, empathy, and tangibles. Data were collected researched from database (PubMed, Embase|, Cochrane ), government reports, and publicly available documents from sources such as the Irish Health Service Executive and the author’s firsthand experience working in Ireland’s healthcare sector.

**Results:**

Key findings highlight Ireland’s success in preserving older adults’ autonomy, reducing informal caregiving pressures, and alleviating financial burdens through standardized care protocols, professional staff training, effective service placement, state financial support, and rapid emergency responses. Notably, competitive compensation and clear career progression pathways for caregivers enhance job satisfaction and retention.

**Conclusion:**

These elements collectively contribute to the high-quality delivery of home-based elderly care services in Ireland.The study suggests China can improve its HSS by adopting similar strategies, including a localized standardized care protocols, caregiver certification, and government-supported financial and career incentives, to strengthen home-based elderly care.

